# Sphingosine-1-Phosphate Metabolism in the Regulation of Obesity/Type 2 Diabetes

**DOI:** 10.3390/cells9071682

**Published:** 2020-07-13

**Authors:** Jeanne Guitton, Cecile L. Bandet, Mohamed L. Mariko, Sophie Tan-Chen, Olivier Bourron, Yacir Benomar, Eric Hajduch, Hervé Le Stunff

**Affiliations:** 1 Institut des Neurosciences Paris-Saclay, Université Paris Saclay, CNRS UMR 9197, F-91190 Orsay, France; Jeanne.guitton@u-psud.fr (J.G.); mohamed.mariko@universite-paris-saclay.fr (M.L.M.); yacir.benomar@universite-paris-saclay.fr (Y.B.); 2 Centre de Recherche des Cordeliers, INSERM, Sorbonne Université, Université de Paris, F-75006 Paris, France; cecile.bandet@gmail.com (C.L.B.); sophie.tan.crc@gmail.com (S.T.-C.); olivier.bourron@aphp.fr (O.B.); eric.hajduch@crc.jussieu.fr (E.H.); 3 Institut Hospitalo-Universitaire ICAN, F-75013 Paris, France; 4 Assistance Publique-Hôpitaux de Paris, Département de Diabétologie et Maladies métaboliques, Hôpital Pitié-Salpêtrière, F-75013 Paris, France

**Keywords:** Sphingosine-1-phosphate, obesity, type 2 diabetes, insulin resistance, pancreatic β cell fate, hypothalamus

## Abstract

Obesity is a pathophysiological condition where excess free fatty acids (FFA) target and promote the dysfunctioning of insulin sensitive tissues and of pancreatic β cells. This leads to the dysregulation of glucose homeostasis, which culminates in the onset of type 2 diabetes (T2D). FFA, which accumulate in these tissues, are metabolized as lipid derivatives such as ceramide, and the ectopic accumulation of the latter has been shown to lead to lipotoxicity. Ceramide is an active lipid that inhibits the insulin signaling pathway as well as inducing pancreatic β cell death. In mammals, ceramide is a key lipid intermediate for sphingolipid metabolism as is sphingosine-1-phosphate (S1P). S1P levels have also been associated with the development of obesity and T2D. In this review, the current knowledge on S1P metabolism in regulating insulin signaling in pancreatic β cell fate and in the regulation of feeding by the hypothalamus in the context of obesity and T2D is summarized. It demonstrates that S1P can display opposite effects on insulin sensitive tissues and pancreatic β cells, which depends on its origin or its degradation pathway.

## 1. Introduction

Obesity is a major public health problem, which results in over nutrition that leads to a net-positive energy balance characterized by the storage of excess fat in the subcutaneous and visceral adipose tissues, as well as in ectopic tissues, including skeletal muscles, liver, and pancreatic β cells [1]. In physiological conditions, ingested lipids are usually used as an energy source by most organisms and can be substituted by carbohydrates for ATP production, based on acute changes in nutrient availability and energy requirements [2]. However, in pathophysiological conditions, adipose tissue lipid metabolism becomes dysfunctional, which leads to increased delivery of fatty acids to other peripheral tissues [3]. Increased free fatty acids (FFA) produced from adipose tissue as well as secretion of hormones, cytokines, and pro-inflammatory markers, which are directly linked to obesity, induce reduced glucose uptake in muscle cells and increased hepatic glucose production. These metabolic dysfunctions lead to a glucose overflow in the circulation, which culminates in glucose intolerance and the installation of type 2 diabetes (T2D) [4].

T2D is a serious metabolic condition due to the insufficient secretion of insulin by pancreatic β cells, and due to an inefficient response from the body to secreted insulin. Diabetes is one of the fastest growing global health emergencies of the 21st century. In 2019, the world prevalence of diabetes was estimated as 463 million people, and this number is projected to reach 578 million by 2030, and 700 million by 2045. T2D is also the most common form of diabetes and accounts for 90% of the disease worldwide [5]. T2D is most commonly observed in older adults but is increasingly seen in children and younger adults due to the rise of obesity, physical inactivity, and inappropriate diet.

High levels of circulating FFA are known to induce not only insulin resistance, but also defects in the insulin secretory capacity of β cells, as well as in insulin gene expression [6,7]. The nature of FFA, that is, its degree of saturation and carbon chain length, is one of the critical factors involved in the induction of lipotoxicity, such as inhibition of insulin secretion, β cell apoptosis, and insulin resistance [8,9]. Non-adipose tissue accumulated FFAs are metabolized into lipid derivatives such as ceramides, which, in turn, lead to lipotoxicity in these tissues [10]. In mammalian cells, ceramides are key lipids of sphingolipid metabolism and are widely distributed in cell membranes where they play a crucial structural role. It also has important functions in intracellular signaling, regulation of growth, proliferation, cell migration, apoptosis, and differentiation [11,12,13]. Ceramides consist of a sphingoid long chain base to which a fatty acid is attached via an amide bond. In the context of fatty acid overload, ceramide is mainly produced *de novo* in the endoplasmic reticulum (ER), through different enzymatic reactions [14,15]. It is now clearly established that *de novo* synthetized ceramides are among the most active lipid second messengers, which inhibits the function of some key proteins of the insulin signaling pathway [16,17] and stimulates pancreatic β cell death [18]. Apart from its structural and signaling functions, ceramide is a central lipid intermediate in sphingolipid metabolism. It is a precursor for other bioactive sphingolipids, from complex glycosphingolipids or sphingomyelin to more “simple” lipids such as ceramide-1-phosphate, sphingosine, and sphingosine-1-phosphate (S1P) [12]. 

**Figure 1:**
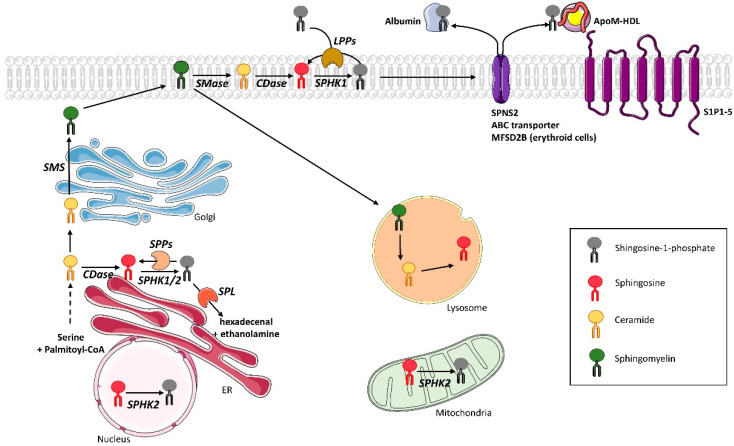
Sphingosine-1-phosphate metabolism in mammals. Sphingolipid *de novo* synthesis is initiated in the endoplasmic reticulum (ER), starting by the condensation of serine and palmitoyl-coA followed by a cascade of enzymatic reactions to produce ceramide. In the ER, ceramide is deacylated by neutral CDase into sphingosine. Sphingosine is phosphorylated to produce S1P by SphK1/2. Produced S1P can be either dephosphorylated back to sphingosine by ER resident SPPs, or irreversibly transformed into hexadecenal and phosphoethanolamine by S1P lyase. Ceramide is transported to the Golgi apparatus to be transformed into SM, which will reach the plasma membrane. In the plasma membrane, SM can be transformed into ceramide through the action of SMases. Ceramide will then be deacylated by acidic CDase to give sphingosine that will be phosphorylated into S1P by SphK1. Produced S1P can be dephosphorylated by ecto-LPPs. S1P can also be secreted through ABC, SPNS2, and MFSD2B transporters in extracellular space to activate S1P receptors. Extracellular S1P can also be transported by either albumin or ApoM/HDL. The latter can activate S1P receptors. SM can be endocytosed to be recycled into ceramide and sphingosine inside lysosomes. SphK2 can catalyze S1P production in the mitochondria and the nucleus. ABC: ATP-binding cassette. CDase: ceramidase. ER: endoplasmic reticulum. HDL: high density lipoproteins. MFSD2B: Major Facilitator Superfamily Domain Containing 2B. S1P: sphingosine-1-phosphate. SM: sphingomyelin. SMase: sphingomyelinase. SphK: sphingosine kinase. S1P1-5: S1P receptor 1 to 5. SPNS2: Spinster homolog 2. SPP: Sphingosine-1-phosphate phosphohydrolase.

The most important site of S1P production is the plasma membrane where sphingomyelin is metabolized into ceramide by sphingomyelinase, then S1P is produced by cooperating two enzyme families, namely ceramidases, and sphingosine kinases (SphK) (Figure 1) [19]. Contrary to most sphingolipids, S1P does not possess a structural function, but is a potent signal mediator that modulates multiple cellular functions important for health and diseases [14]. The multimodal actions of S1P can be explained by the fact that the sphingolipid, on the one hand, directly modulates intracellular functions, and, on the other hand, acts as a ligand of G protein-coupled receptors (GPCR) after secretion into the extracellular environment, transported by ApoM-containing high density lipoproteins (HDL) or albumin, to exert either autocrine and/or paracrine functions [19]. 

In many cellular and animal models, both ceramide and S1P display opposite effects. This is well documented in cancer cells where ceramide stimulates apoptosis, whereas S1P promotes cell survival [11]. While the role of ceramide in the development of muscle insulin resistance is now well established [16], the relationship of S1P with insulin resistance and T2D still remains controversial in some tissues. Elevation of tissue and plasma S1P levels has been recognized as a critical feature of both human and rodent obesity [20], which suggests that S1P metabolism could be involved in the onset of T2D, or that its regulation is an adaptive process in the presence of high levels of circulating lipids. Thus, this review describes the role played by S1P metabolism in the development of obesity/T2D by analysing the enzymes regulating both its tissue and circulating levels in insulin resistance of peripheral tissues and pancreatic β cell fate.

## 2. S1P Metabolism in Mammals

### 2.1. S1P Synthesis

S1P is produced by deacylation of ceramide by ceramidases to give sphingosine. Subsequently, sphingosine kinases (SphK) are responsible for the phosphorylation of sphingosine, which results in the formation of S1P (Figure 1). As to the anabolic pathway of S1P, two isoforms of sphingosine kinases (SphK) have been discovered, called SphK1 and SphK2. Both are widely expressed [21]. Compared to SphK1, SphK2 possesses 240 additional amino acids in its N-terminal region corresponding to a nuclear export sequence [22]. Although they have similar sequences, these enzymes differ in their intracellular localization, regulation, level of tissue expression, and, therefore, in their functions [23], especially in sphingolipid metabolism and, thus, the level of ceramide [24].

While SphK1 resides in the cytosol, SphK2 is localized in the nucleus, the inner mitochondrial membrane, and the endoplasmic reticulum (ER) (Figure 1). Under basal conditions, SphK1 is mostly present in the cytoplasm. SphK1 catalytic activity increases from 1.5 to 4-fold as it translocates to the plasma membrane upon stimulation. Both translocation and activity are regulated not only by the phosphorylation of SphK1 Ser^225^ residue by extracellular signal-regulated kinases (ERK1/2) [25], but also by anionic lipids (phosphatidylserine and phosphatidic acid) and Ca^2+^/calmodulin [22].

SphK2 can also be phosphorylated by ERK1/2, but the exact phosphorylation site remains unclear, as Ser351 and/or Thr578 residues may be involved [25]. As SphK2 is localized in the nucleus, it can directly interact and form a complex with H3 histone and histone deacetylases 1 and 2 (HDAC1/2) in the promotor of transcriptional regulator c-fos and dependent kinase inhibitor p21 genes, where it enhances local histone H3 acetylation and transcription [26]. Synthetized S1P by SphK2 binds to and inhibits both HDAC1 and HDAC2, which suggests that nucleus-generated S1P via SphK2 influences the dynamic balance of histone acetylation and, thus, the epigenetic modulation of specific target genes [27]. In addition, when produced in the mitochondria by SphK2, S1P regulates prohibitin 2 (PHB2) function, which is a highly conserved protein that regulates mitochondrial homeostasis [28].

According to Maceyka et al., SphK1 and SphK2 display opposite functions in sphingolipid metabolism in the regulation of ceramide biosynthesis. Indeed, in HEK293 cells, specific down-regulation of SphK2 reduced conversion of sphingosine into ceramide in the recycling pathway and, conversely, down-regulation of SphK1 increased it [24]. This difference could be linked to a potent dialogue between SphK2 and the S1P phosphatase 1 (SPP1) that favors the conversion of S1P into ceramide [29] (see below Section 2.2).

### 2.2. S1P Recycling and Degradation

S1P can be quickly and irreversibly degraded by the endoplasmic reticulum resident enzyme S1P lyase (SPL), which cleaves the C2-C3 bond of S1P to generate two products: hexadecenal (palmitaldehyde) and phosphoethanolamine [30] (Figure 1). Both products can then be transferred as glycerol substrates and phospholipid substrates in the glycerophospholipid pathway [31]. Phosphoethanolamine will be used for the synthesis of phosphatidylethanolamine and hexadecenal will be used for reloading the palmitoyl-CoA pool [31].

Alternatively, S1P can also be reversibly dephosphorylated by several phosphohydrolases to regenerate sphingosine. The first lipid phosphohydrolases involved are lipid phosphate phosphohydrolases (LPPs) (Figure 1). They belong to the superfamily of lipid phosphatases that includes three isoforms characterized in mammals: LPP1, LPP2, and LPP3. LPPs are membrane-associated, magnesium-independent and N-ethylmaleimide-insensitive enzymes [29]. Their active sites are located on the outer surface of plasma membranes or at the lumenal surface of internal membranes (Golgi and endosomes) [32]. LPP2 resides intracellularly, whereas LPP1 and LPP3 are mainly localized at the plasma membrane and function as ecto-enzymes, while degrading lipid phosphate substrates such as S1P as well as lysophosphatidic acid in the extracellular space [33].

S1P can also be dephosphorylated by two specific S1P phosphohydrolases called SPP1 and SPP2 (Figure 1). These two mammalian isoforms are differentially expressed-sphingoid base-specific phosphatases localized in the ER. SPP1 regulates the salvage of sphingosine for the synthesis of ceramide in the ER (rescue pathway) [33], and it has been shown that SPP1 overexpression induces ceramide accumulation in the ER, which suggests that dephosphorylation of S1P is a limiting step for the recycling pathway [33]. A regulatory role in the recycling pathway for SPP2 has not yet been demonstrated, but its expression was increased during the inflammatory response [33]. In addition, it was reported that both SPP1 and SPP2 were also involved in ER stress-induced-autophagy [34] and proliferation [35].

### 2.3. S1P Transport

Contrary to most sphingolipids, S1P does not possess any structural function, but is a potent signal mediator that affects multiple cellular functions important for health and diseases. The multitude of different S1P-mediated actions is linked to its capacity to be secreted by various cells and tissues. To exert its extracellular functions, intracellularly generated S1P is transported across the plasma membrane. Since S1P is too hydrophilic to simply diffuse through the membrane, it is exported by specific ATP-binding cassette (ABC) transporters or the spinster homolog 2 (SPNS2) transporter, which is a member of non-ATP-dependent organic ion transporter family [36]. In the erythrocyte, S1P was recently shown to be secreted through the protein MFSD2B [37]. Once outside the cell, S1P can either bind to albumin [38], or ApoM [39]. Approximately 35% of plasma S1P is bound to albumin and 65% to ApoM, which is found on a small percentage (~5%) of high density lipoprotein (HDL) particles [40]. S1P has a four-times longer half-life when bound to ApoM/HDL than to albumin, as seen when tested in vivo (15 min) and in vitro (30 min) under albumin binding conditions [41,42]). This suggests that the binding of S1P to HDL prevents its degradation. ApoM/HDL-bound S1P has been proposed as a primary contributor to the vasoprotective properties of HDLs [43], and S1P has also been shown to be a key component in the anti-atherogenic properties of HDL [44]. However, S1P-bound albumin has been suggested to represent a reservoir for free S1P [39].

### 2.4. S1P Receptors

As an extracellular second messenger, S1P is a high-affinity ligand (Kd from 2 to 63 nM) of a family of five GPCRs, termed S1P1-5 [45]. Receptor-bound S1P induces a wide range of physiological responses such as proliferation, migration, inhibition of apoptosis, formation of actin stress fibers, stimulation of adherent junctions, and enhanced extracellular matrix assembly [46]. S1P1–3 are ubiquitously expressed throughout tissues, whereas S1P4 is predominantly expressed in the immune system, and S1P5 is expressed in the central nervous system and the spleen [27]. S1P receptor activation on different cell types depends on specific G protein coupling. S1P1 couples exclusively with the inhibitory G protein alpha subunit (Gαi), whereas S1P2 and S1P3 bind to Gαi, Gαq, and Gα13, while S1P4 and S1P5 couple to both Gαi and Gα13 [47]. Following ligand binding and subsequent activation, the α subunit of the heterotrimeric G protein is released and interacts with various downstream effectors (see review [48] for more information).

## 3. S1P Metabolism and Insulin Action: Muscle, Liver, and Adipose Tissue

Since the early 2000s, several studies have looked for the potential role of S1P in mediating insulin action in insulin-sensitive tissues such as liver, skeletal muscle, and adipose tissue.

### 3.1. Liver

Liver is a major organ for glucose and lipid metabolism, and it has been known for many years that lipid accumulation is linked to the development of insulin resistance and constitutes the first stage of non-alcoholic fatty liver diseases (NAFLD) [49]. Several studies have shown that the SphK1/S1P axis can control the insulin response in the liver. One such study highlighted that hepatic SphK1 expression increased in animals under lipid overload induced by a high-fat diet (HFD) [40]. This increased expression of SphK1 was also found in the liver of human patients displaying NAFLD [40].

Several other studies have also highlighted a positive action of the SphK1/S1P axis on glucose metabolism in hepatocytes. A pioneer study performed in human hepatocytes showed that tumor necrosis factor α (TNFα), which is a cytokine involved in inflammation [50], was unable to activate the NFκB pathway-induced apoptosis, but rather activated the pro-survival SphK1 pathway [51]. The authors also emphasized that TNFα protected hepatocytes from apoptosis by activating SphK1 upstream of the PI3K/Akt pathway [51]. An increase in Akt phosphorylation was observed after 5 min of treatment with exogenous S1P (without insulin), which suggests that a relationship between the SphK/S1P axis and the PI3K/Akt pathway exists in hepatocytes [51]. Similar results were observed by treating primary rat hepatocytes with exogenous S1P [52]. In addition, treatment of a human liver cell line (LO2 cells) with S1P induced an increase in glucose uptake [53]. SphK1 overexpression also induced glucose uptake in the absence of insulin in hepatocellular carcinoma [54]. Conversely, inhibition of SphK1 reduced glucose uptake in the presence or absence of insulin in the same cell line [54]. The authors extended their observation in vivo by injecting an adenoviral vector containing the human SphK1 cDNA in diabetic KK/Ay mice [54]. Under these conditions, transfected diabetic KK/Ay mice displayed a decrease in basal glycemia and a better glucose tolerance compared to control animals [54]. In parallel, they observed a decrease in total cholesterol, triglycerides, and low density lipoproteins as well as an increase in circulating HDL in SphK1-transfected animals compared to control animals [54].

**Figure 2:**
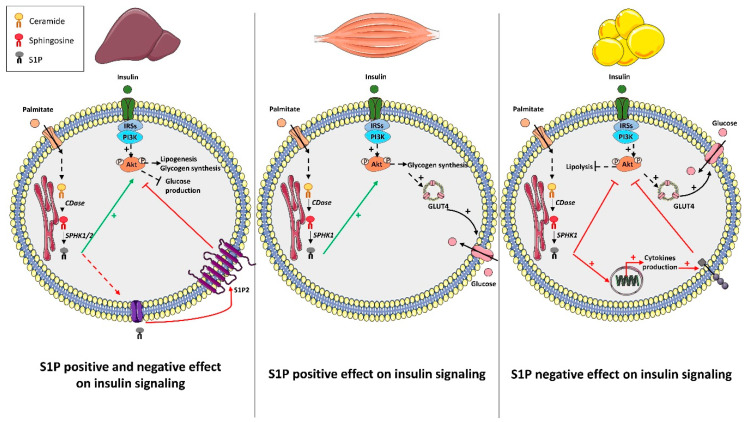
Role of S1P metabolism on insulin in peripheral tissues in response to palmitate. In hepatocytes, palmitate increases intracellular S1P content through SphK1/2 activities. According to studies, produced S1P seems to exert a direct positive action on insulin signaling, or a negative action by stimulating its S1P2 receptors. In muscle cells, palmitate increased intracellular S1P through SphK1 activity, which favors Akt activation, glucose uptake, and glycogen synthesis in response to insulin. In adipocytes, palmitate increases intracellular S1P to inhibit Akt activation in response to insulin. Produced S1P also favors expression of pro-inflammatory cytokines that will contribute to inhibit Akt activity. CDase: ceramidase. GLUT4: glucose transporter 4. IRS: insulin receptor substrate. PI3K: phosphatidylinositol-3-kinase. SphK: sphingosine kinase. S1P2: S1P receptor 2.

All of these parameters demonstrated that SphK1 overexpression in diabetic animals improved glucose homeostasis at the systemic level. The authors also assessed the hepatic insulin response in these animals, and they showed that both Akt and GSK3 phosphorylation levels were increased in animals overexpressing SphK1 when compared to control animals (Figure 2) [54].

These results were confirmed in another study showing that increased mouse hepatic S1P following the overexpression of acid sphingomyelinase (ASM) favored hepatic Akt phosphorylation as well as improved glucose tolerance [55]. When SphK1 expression was reduced in these animals, increased Akt phosphorylation was no longer observed (Figure 2) [55]. In the same study, these observations were confirmed in vitro by treating isolated primary hepatocytes with exogenous S1P, which induced an increase in Akt phosphorylation in the absence of insulin [55].

Contrary to SphK1 expression, incubation of primary mouse hepatocytes with palmitate did not induce any increase in SphK2 expression [56]. Nonetheless, a hepatic role of SphK2-produced S1P in regulating glucose metabolism was investigated by Lee et al. [56]. Endoplasmic reticulum (ER) stress is known to participate in the development of insulin resistance in liver, mainly by promoting the accumulation of lipids in the liver, by directly blocking insulin signaling, and by modifying the expression of key enzymes of gluconeogenesis or lipolysis [49]. Lee et al. found that ER stress transcriptionally up-regulated SphK2 in liver [56]. Overexpression of SphK2 in the AML12 hepatocyte cell line induced an increase in S1P concentration, which was associated with increased Akt phosphorylation in the absence of insulin [57]. In addition, SphK2 overexpression induced a decrease of some sphingolipid species (C16-ceramide, C18-ceramide, C18:1-ceramide, C16-sphingomyelin, C18-sphingomyelin) as well as a decrease in cholesterol and hepatic triglyceride concentration [57]. As observed previously in SphK1-overexpressing animals, hepatic SphK2 overexpression induced an improvement in insulin sensitivity, an increase in hepatic Akt phosphorylation, and, therefore, an improvement in glucose tolerance of these animals when fed an HFD (Figure 2) [57].

Although these studies demonstrate that the hepatic SphKs/S1P axis positively regulates liver insulin response and carbohydrate metabolism under lipotoxic conditions, some other studies showed the opposite and gave a deleterious role to this axis of hepatic insulin signaling. One study reported that S1P inhibited insulin signaling in the liver both in vitro and in vivo [58]. As already described above, Fayyaz et al. showed that, after palmitate treatment, concentrations of intra- and extracellular S1P were increased in primary rat hepatocytes [58]. However, they also observed generated S1P counteracted insulin signaling [58]. The negative role of S1P on insulin signaling in rat or human hepatocytes with exogenous S1P was counteracted in the presence of JTE-013, which is an S1P2 antagonist. This suggests that S1P inhibited the insulin signal through the activation of S1P2 receptor [58]. These observations were extended in vivo Diabetic New Zealand obese (NZO) mice were treated with JTE-013 for seven days before being sacrificed. Both an increase in liver Akt phosphorylation and a decrease in basal glycaemia were observed [58]. Overall, this study demonstrates that palmitate-produced S1P stimulates S1P2 to impair hepatocyte insulin signaling (Figure 2).

In addition, other studies have also highlighted a relationship between liver S1P levels and hepatic lipid accumulation. Mouse hepatic overexpression of ASM has been shown to increase hepatic triglyceride content, which was blunted by SphK1 deletion [55]. This suggests a potent role of SphK1 in steatosis. SphK1 knock-out (KO) mice fed an HFD for 24 weeks displayed an increase in circulating triglycerides compared to wild-type (WT) animals fed the same diet [59]. By contrast, mice displaying a liver-specific overexpression of SphK1 via the use of an adeno-associated-viral (AAV) 8, whose tropism is specific of the liver [60], exhibited reduced hepatic triglyceride levels (steatosis) without affecting glucose metabolism on a low-fat diet [60]. However, no impact of increasing SphK activity on hepatic lipid content or glucose metabolism was observed in HFD fed mice [60]. The discrepancies between these studies could arise from animal models, which use enzyme overexpression (i.e., ASM and SphK1). Lastly, a study showed that SphK1 expression increased in hepatic steatosis and that SphK1 KO mice were protected against hepatic steatosis induced by HFD [56], which supports the idea that endogenous S1P/SphK1 axis could be a major promoter of lipid accumulation in liver (steatosis) [61]. In contrast, SphK2 KO mice fed with HFD showed an increase in hepatic lipid accumulation, which supports the idea that this isoform protected mice from steatosis [62].

Overall, the effect of the SphK/S1P axis on liver glucose metabolism remains not completely solved. Most of the genetic approaches used, to either overexpress or invalidate SphK1 (and SphK2), showed a positive action of the SphK/S1P axis on hepatic insulin response. However, these studies were carried out at the level of the whole organism and, thus, were not liver-specific. It is, therefore, possible that, in addition to hepatic S1P, circulating S1P coming from other tissues could also affect hepatic homeostasis. It has already been shown that hepatic S1P could be secreted to regulate macrophage chemotaxis [63]. The divergent effect of hepatic S1P could also be related to the specific activation of S1P receptors [58] and will require more exploration as to their role in liver homeostasis during obesity. Moreover, it also remains to determine how S1P signals could move from the beneficial effect through insulin signaling to the dysregulation of lipid homeostasis (steatosis). Only one clinical study has shown SphK1 expression increase in liver biopsies from patients with steatosis compared to healthy lean people, which supports the notion that SphK1/S1P axis could play a role in the onset of these diseases [64]. However, whether the localization of increased SphK1 in the human liver is specific just to hepatocyte, as well as its role, still remain unknown. Therefore, future work and analysis will be required to translate data obtained in cell/mouse to those in humans.

### 3.2. Muscle

Muscles constitute 40% of the body weight and are responsible for 40–75% of the glucose uptake in response to insulin in the postprandial period [65]. They are, therefore, major tissues toward the regulation of carbohydrate homeostasis within the body. Compared to liver, few studies have looked for the role of S1P on glucose metabolism in muscle, but, unlike liver, it seems that they all demonstrate a positive action of this lipid.

Saturated fatty acid (palmitate) induced an increase in SphK1 expression as well as an increase in S1P concentrations in a muscle cells line (C2C12 myotubes) [66], and in mouse primary myotubes [67]. It is important to note that no increase in SphK1 expression was observed in response to unsaturated fatty acids such as oleate [66]. These data were confirmed in vivo where a 2.5-fold increase in SphK1 expression was observed in skeletal muscles of mice fed an HFD compared to control mice (Figure 2) [66]. The addition of exogenous S1P on C2C12 myotubes increased basal Akt phosphorylation, which led to a concomitant increase in glucose uptake [68]. 

In vivo studies also reported a positive role of SphK1/S1P on insulin signaling in muscle. SphK1-overexpressing mice displayed increased SphK activity in skeletal muscle, and when fed a HFD, skeletal muscle and whole-body insulin sensitivity were improved in these mice compared with control mice fed the same diet [69]. In addition, animals overexpressing SphK1 fed an HFD for six weeks displayed better muscle Akt phosphorylation and were more glucose-tolerant and more sensitive to insulin than wild type animals (Figure 2). However, although skeletal muscles show an increase in SphK1 overexpression, it cannot be excluded that other untested tissues could also overexpress SphK1 and, thus, participate with the observed phenotype.

To complicate the picture, Bruce et al. showed that SphK1 overexpression induced a decrease in muscle ceramide concentration [69]. Considering the importance of this sphingolipid species in the development of insulin resistance [66,67], it remains difficult to ascertain if the observed phenotypes were linked to a decrease in ceramide content or rather from an independent action of S1P. Likewise, both Bruce et al. [70] and Kendall et al. [71] showed that administration of FTY720, which is an S1P analogue that downregulates all S1PR expressions except for S1P2 [72], to animals fed an HFD induced a better muscle insulin signaling as well as a better glucose tolerance compared to animals receiving vehicle only. However, FTY720, which has also been shown as a potent inhibitor of CerS [73], inhibited ceramide production in mice under HFD [70]. These data suggest that the insulin sensitizer effect of FTY720 was associated with a decrease of ceramide levels in muscle rather than an antagonist action on S1P receptors.

Altogether, even if all studies reported a positive role of the SphK1/S1P axis on muscle insulin signaling, and, consequently, on the systemic glucose metabolism, no specific muscle approach was performed. Thus, this possibly hid some cross-talk mediated by S1P between muscles and other S1P producing tissues such as liver, adipose tissue, or even immune cells. In addition, no study, so far, has investigated the role of SphK2-produced S1P in this tissue, nor shown the opposite roles of SphK1 and SphK2 [24]. However, it would be interesting to study the role of the latter on insulin signaling in muscle. It would also be important to explore the role of S1P catabolism and S1P signaling through its receptors in muscle homeostasis. To date, clinical data demonstrating the role of S1P metabolism in regulating muscle insulin resistance in man are lacking and will, therefore, require extensive study.

### 3.3. Adipose Tissue

Adipose tissue (AT), in addition to its storage functions, is an endocrine tissue that secretes several adipokines and chemokines [74]. AT also participates in the development of insulin resistance when it is in a state of inflammation known as “low-grade” [75]. In addition, when maximum AT storage capacities are reached, excess lipids are then stored in peripheral tissues, which causes insulin resistance or apoptosis in these various tissues [16]. Homeostasis of adipose tissue is, therefore, important for maintaining sensitivity to systemic insulin [76].

Expression of SphK1, but not SphK2, has been reported to be increased in subcutaneous adipose tissue from *ob/ob* mice compared to wild type mice [77]. Similar results were observed in epididymal adipose tissue and isolated mature adipocytes from mice fed an HFD compared to animals on a low-fat diet [78]. Similar profiles were also reported in human inflamed subcutaneous AT compared to less inflamed AT [58]. Concentrations of S1P are also increased in subcutaneous AT from obese patients compared to those from lean people [79].

One study reported a positive role of S1P on insulin signaling in AT. Administration of the S1P analogue FTY720 improved insulin sensitivity in animals fed an HFD [71]. Immune cell infiltration is known to play an important role in insulin resistance [80], and it was found that FTY720 decreased lymphocyte and macrophage infiltration in TA of this mice, likely through its lymphopenic properties [81]. This phenomenon contributes to improving insulin sensitivity in mice. However, another study demonstrated the opposite results. It showed that, in HFD-fed mice, SphK1 deficiency increased adipogenic markers such as adiponectin and the anti-inflammatory cytokine IL-10, but reduced adipose tissue macrophage recruitment as well as pro-inflammatory molecules TNFα and IL-6 (Figure 2). These changes were associated with a better insulin response in the AT and improved insulin sensitivity and glucose tolerance (Figure 2) [78]. Obesity was found to increase SphK1 expression in AT macrophages of both M1 and M2 phenotypes [82]. Elevated SphK1 expression in AT macrophages was associated with the reduction of endoplasmic reticulum stress related genes, which suggests that Sphk1 promotes AT macrophage survival [82].

Overall, these few studies indicate that SphK1/S1P axis leans towards a pro-inflammatory and negative action on AT insulin signaling. However, extracellular S1P through S1P receptors may have the opposite effect [71]. Therefore, analysis of the role of other S1P metabolic enzymes in adipose tissue homeostasis will be necessary to confirm this tendency. It will also be important to decipher whether differences in S1P function exist between AT distributions (visceral vs. subcutaneous) known to play a distinct role in obesity. Although SphK1 expression is increased in the adipose tissue from obese patients [78], no clinical study has, so far, described the functional role of S1P in human adipocyte insulin resistance.

## 4. S1P Metabolism and Pancreatic β Cell Fate

Pancreatic β cells secrete insulin in response to glucose and various hormones to maintain glycaemia and, therefore, regulate glucose homeostasis. However, obesity is associated with deleterious effects of elevated fatty acid levels on pancreatic β cell function and survival. Excessive fatty acids leads to the loss of β cell insulin secretory responsiveness and β cell death by apoptosis, which favors induction of chronic hyperglycemia [18]. Sphingolipids and, in particular, ceramide have been shown to play a central role in pancreatic β-cell apoptosis induced by palmitate [18]. More recently, S1P has also been implicated in mediating β-cell function and viability with a specific role for its metabolizing enzymes. 

**Figure 3:**
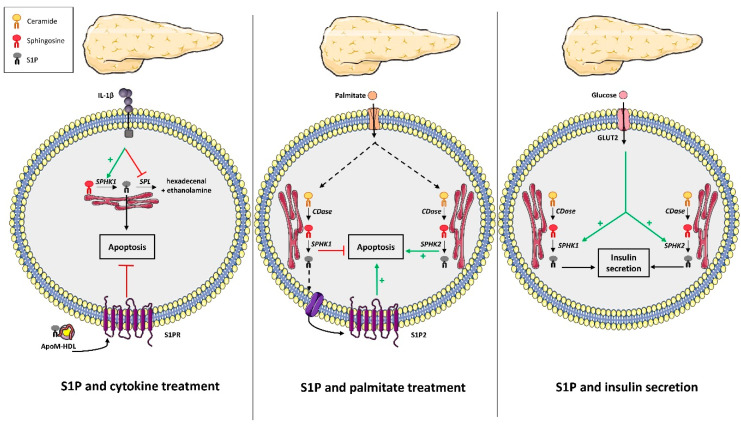
Role of S1P metabolism on pancreatic β cell fate. In pancreatic β cells, cytokines, such as IL1β increase SphK1 expression and repress SPL expression. This contributes to the increase of intracellular S1P content and apoptosis. In contrast, extracellular ApoM/HDL-bound S1P y antagonizes apoptosis induced by IL1β. In pancreatic β cells, palmitate increases the expression of both SphK1 and2. SphK1 activation represses palmitate-induced pancreatic β cell apoptosis, whereas SphK2 activation promotes apoptosis. SphK1-produced S1P can be secreted and stimulates S1P2 to promote apoptosis. High glucose levels could activate both SphK1 and 2, which contribute to the secretion of insulin. CDase: ceramidase. SPL: S1P lyase. SphK: sphingosine kinase. S1P2: S1P receptor 2.

In 2005, a pioneering study characterized the SphK/S1P axis in rat pancreatic β cells and in INS-1 cells [83]. This study was followed by numerous others that focused on the SphK/S1P axis involvement in β-cell secretory function. Hasan et al. reported for the first time that SphK1 activity was important for insulin synthesis and secretion [84]. The knock-down of SphK1 expression in pancreatic β INS-1 cells resulted in both lowered glucose-stimulated insulin secretion (GSIS) and insulin content associated with decreased insulin gene expression. Conversely, SphK1 overexpression restored both insulin synthesis and secretion [84]. In contrast, pancreatic β MIN6 cells exposed to high glucose concentrations displayed an increase in S1P levels due to SphK2 activity, which is concomitant with higher insulin secretion. In addition, inhibition of S1P production through SphK2 KO in MIN6 cells resulted in the abolition of GSIS [85]. Overall, these data suggest that S1P synthesis through both SphK1 and SphK2 could be positively involved in regulating insulin secretion (Figure 3). However, this conclusion still needs in vivo and in vitro exploration of GSIS in mice KO for either SphK1 or SphK2.

The SphK/S1P axis was shown to be stimulated by cytokines in rat pancreatic β cells and INS-1 cells (Figure 3) [83], which suggests a potential role in the pathological response to cytokines observed during low-grade inflammation induced by obesity. Later on, Hahn et al. showed that cytokines decreased SPL expression in pancreatic β cells, whereas overexpression of SPL protected them against cytokine toxicity (Figure 3) [86], which comforts a pathological role of intracellular S1P metabolism of pancreatic β cells in diabetes. In contrast, Laychock et al. showed that exogenous S1P counteracted pancreatic β cell apoptosis induced by cytokines [87], which suggests a divergent role of cellular S1P from circulating S1P (Figure 3). Supporting this notion, Rütti et al. found that HDL, known to be enriched in S1P through its binding to apoM, also counteracted pancreatic β cell apoptosis induced by cytokines (Figure 3) [88].

Although the SphK/S1P axis appears to regulate β-cell induced-apoptosis induced by cytokines, the circulating levels are increased by obesity, whether it is implicated in β-cell apoptosis induced by free fatty acids still remains unknown. Palmitate increased not only ceramide but also S1P levels, through SphK1 up-regulation in pancreatic β INS-1 cells (Figure 3) [89]. Japtok et al. also demonstrated that palmitate increased S1P levels in pancreatic β MIN6 cells, which were released in the extracellular medium (Figure 3) [90]. Apoptosis was abrogated in INS-1 cells over-expressing SphK1 [89]. Similarly, either S1P supplementation or SphK1 overexpression in palmitate-treated INS-1 or MIN6 cells prevented cell death (Figure 3) [59]. Conversely, dominant negative expression of SphK1 in these cell lines enhanced palmitate-induced apoptosis [59]. The protective role of SphK1 was independent of S1P receptors, but was mediated by decreasing formation of pro-apoptotic ceramides induced by palmitate [89]. In addition, endoplasmic reticulum-targeted SphK1 also partially inhibited apoptosis induced by lipotoxicity, which suggests a specific localization for the anti-apoptotic action of S1P [89]. Nevertheless, JTE-013, which is an antagonist of S1P2, partially counteracted pancreatic β-cell apoptosis and the reduced proliferation induced by palmitate [89,90], which suggests that the S1P produced could determine pancreatic β-cell fate under lipotoxicity by interacting with specific receptors (Figure 3). Overall, these studies reported a survival and protective role of both intracellular S1P and its enzyme SphK1 against palmitate-induced β-cell apoptosis [59,90].

In addition, one study discovered that HFD-fed SphK1 KO mice displayed a reduction in β cell size, number, and mass associated with increased β cell apoptosis compared to WT HFD-fed mice, which all favor the installation of glucose intolerance [59]. These data indicated that in vivo SphK1 deficiency predisposes mice to T2D-onset by promoting pancreatic β cell death under lipotoxic conditions [59]. SphK1 has been shown to interact with SKIP (SPHK1-interacting protein) and that SKIP overexpression in NIH 3T3 fibroblasts reduces SphK1 activity and interferes with its biological functions [91]. In another study, SKIP-deficient mice improved glucose tolerance by increasing insulin and GLP-1 secretion [92], which suggests that SKIP deficiency in mice allow SphK1 to better regulate glucose tolerance. However, it remains to be established whether SKIP is acting only at the level of intestinal L cells or on the pancreatic β cell since it is already known that islet-derived GLP-1 is necessary for glucose-stimulated insulin secretion [93]. Not surprisingly, a consensus on the role of the SphK1/S1P axis in β-cells has not been reached. Although the above studies demonstrated a beneficial role of SphK1 on glucose homeostasis and β cell function, another study showed that SphK1 KO mice were protected from HFD-induced glucose intolerance due to a reduced adipocyte pro-inflammatory response, which suggests a negative role of SphK1/S1P axis on regulating glucose homeostasis [78].

In contrast, a negative role of the SphK2/S1P axis was observed on β-cell fate. SphK2 expression KO reversed palmitate-induced cell death, whereas SphK2 overexpression promoted cell death under lipotoxic conditions in both INS-1 and MIN6 cells (Figure 3) [94]. In fact, lipotoxicity induced the shuttling of SphK2 from the nucleus to the cytoplasm, where it led to mitochondrial apoptosis [94]. SphK2 KO diabetic mice under HFD significantly improved their diabetic phenotypes [94], which suggests that, contrary to SphK1, SphK2 exerts a major role in promoting lipotoxicity-induced apoptosis of β cells [94]. Mice with a deletion of the S1P phosphohydrylolase SPP2 exhibited glucose intolerance due to a defect in the adaptation of pancreatic β cell mass, which supports the idea that the rise of endogenous S1P regulated by SphK2 and SPP2 can promote β cell lipotoxicity [35].

Overall, the opposed functions on β cell survival between both SphKs could be explained by expression differences observed in pathophysiological situations but more likely by differences in produced S1P subcellular localization. Nevertheless, it remains crucial to determine the potent role of S1P receptors in pancreatic β cell fate during obesity. To date, there are no clinical studies available describing a potential role of S1P in human islets in the context of obesity or T2D.

## 5. S1P and the Hypothalamic Regulation of Body Weight and Energy Homeostasis

The hypothalamus is a key brain area that plays a crucial role in regulating energy metabolism. It consists of several nuclei including the arcuate nucleus (ARC), ventromedial (VMH), dorsomedial (DMH), lateral (LH), and paraventricular (PVH) hypothalamus, which interact functionally to coordinate adaptive physiological responses controlling feeding behavior and energy expenditure. This process involves the integration of metabolic, endocrine, and neural signals from the periphery and autonomic circuitries that encode information about energy availability and energy reserve in the body [95,96,97,98].

Growing evidence suggests that hypothalamic lipid sensing plays a key role in controlling food intake, fat deposition, and energy balance [99,100], and that its dysregulation could lead to the development of obesity and T2D. Recent investigations reported that S1P is involved in the hypothalamic control of energy homeostasis [101]. Precisely, the intracerebroventricular (ICV) administration of S1P decreased food intake and increased energy expenditure [101]. Conversely, selective disruption of S1P1 in the mediobasal hypothalamus (MBH) induced the opposite effects [101]. At the molecular level, S1P exerted its effects by activating S1P1, which is highly expressed in key hypothalamic nuclei, ARC, VMH, and DMH, which controls feeding, particularly in the anorectic pro-opiomelanocortin (POMC) neurons of the ARC. Altogether, these findings identified S1P/S1P1/JAK2/STAT3 as a new regulatory pathway that plays a crucial role in the hypothalamic control of energy homeostasis and body weight gain. A positive correlation between plasma S1P and body fat percentage exists [20,102], as rodent models of obesity also exhibited an increased hypothalamic S1P/S1P1/STAT3 signaling [101,103]. From a therapeutic point of view, the ICV injection of S1P or the S1P1 agonist, SEW2871, induced anorexigenic effects, and prevented the development of obesity and associated metabolic dysfunctions [101].

In the context of an HFD-induced obesity, and, as it has already been observed in the AT (see section on adipose tissue), inflammatory processes also occur in the brain [104]. HFD triggers brain inflammation, notably in the hypothalamus, by activating microglia and astrocytes, which results in reactive gliosis, production of pro-inflammatory cytokines such as IL1β and TNF, and the development of neuronal inflammation [105]. This contributes to the deregulation of hypothalamic control of energy homeostasis, which promotes the development of obesity and associated metabolic disorders [106,107,108]. Emerging evidence suggests a pivotal role of S1P metabolism and S1P-mediated signaling in the development of neuro-inflammation. It was shown that S1P was able to induce astrocytes activation [109,110] and increase the inflammatory response of activated microglia, which results in reactive gliosis and the upregulation of pro-inflammatory cytokines [111,112]. Additionally, S1P modulated neuro-inflammation by regulating the infiltration of peripheral immune cells in the central nervous system [113,114]. Considering the emerging importance of S1P metabolism in neuro-inflammation, further studies will be required to determine the role of S1P signaling in the early onset of hypothalamic inflammation and gliosis in the context of diet-induced obesity.

The role of hypothalamic S1P in the regulation of obesity and dysregulation of glucose homeostasis is actually an emerging area. Thus, future studies will be important to determine the role of S1P metabolism and signaling at the level of the hypothalamus in the context of obesity and T2D. This will constitute an important step toward identifying new targets for therapeutic intervention in obesity and obesity-related metabolic disorders.

## 6. Conclusions and Perspectives

The elevation of S1P levels in tissues and plasma has been associated with obesity, which suggests that S1P metabolism could be negatively or positively linked to this pathology and to the onset of T2D. Many studies performed in the last decade suggest that S1P metabolism plays a positive role in insulin signaling in peripheral tissues, which points to an adaptive role of S1P to counteract the installation of insulin resistance in muscle, adipose tissue, and liver [54,55,57,69]. However, it should be noted that some studies argue for a causative role of S1P metabolism in insulin resistance in the liver and in adipose tissue [58,78]. S1P metabolism has also been linked to pancreatic β-cell fate during obesity or T2D with opposite roles of S1P produced by SphK1 or SphK2 on pancreatic β cell apoptosis [59,94]. Moreover, the specific role of the SphK1/S1P axis in glucose homeostasis will require further attention since studies reveal divergent phenotypes of SphK1 KO mice under HFD [59,78].

These discrepancies in the results may be linked to the fact that cellular S1P levels are fine-tuned by a concerted regulation of S1P synthetizing enzymes (SphK) and S1P degradation enzymes (SPL and SPPs). The other reason could come from the intrinsic nature of S1P, which is both an intracellular mediator and a circulating bioactive lipid. This supports the idea that S1P could act not only intracellularly but also as an endocrine or paracrine signal through its secretion to regulate the insulin response in distant organs as well as in the pancreatic β-cell fate. Plasma apoM/HDL-bound S1P has been shown to regulate brown adipose tissue activity in the context of obesity [115] and also pancreatic β-cell survival induced by cytokines [88].

In conclusion, more work is required to understand the role of the enzymes involved in S1P metabolism/signaling, especially of the catabolizing enzymes SPL and SPPs in the development of obesity and diabetes. It will also be important to determine the role of its transporters and its receptors. Due to the duality of actions of S1P (intracellular and extracellular), the development of tissue-specific disruption of S1P metabolism enzyme genes in mice would also help us understand the divergent roles of S1P observed in whole KO models used to date. This would be crucial before the modulation of S1P metabolism can be considered as a potential therapeutic target for treating obesity/T2D.
